# Adapting Ophthalmology Practices in Puerto Rico During COVID-19: A Cross-Sectional Survey Study

**DOI:** 10.3390/epidemiologia6030042

**Published:** 2025-08-06

**Authors:** Surafuale Hailu, Andrea N. Ponce, Juliana Charak, Hiram Jimenez, Luma Al-Attar

**Affiliations:** 1 College of Medicine, University of Central Florida, Orlando, FL 32827, USA; 2 Johns Hopkins Bloomberg School of Public Health, Baltimore, MD 21205, USA; aponce4@jhu.edu; 3 Retina Center of Puerto Rico, Manati, PR 00674, USA; julianacharak416@gmail.com; 4 Department of Ophthalmology, University of Puerto Rico School of Medicine, Medical Sciences Campus, San Juan, PR 00936, USA; hiram.jimenez@upr.edu

**Keywords:** ophthalmology, COVID-19, Puerto Rico, practice adaptation, telemedicine, public health, personal protective equipment, pandemic response

## Abstract

**Background/Objectives**: The COVID-19 pandemic caused pronounced disorder in healthcare delivery globally, including ophthalmology. Our study explores how ophthalmologists in Puerto Rico (PR) altered their practices during the pandemic, confronting obstacles such as resource shortages, evolving public health mandates, and unique socio-economic and geographic constraints. The study aims to enhance preparedness for future public health crises. **Methods:** We conducted descriptive analyses on four online surveys distributed at crucial time points of the pandemic (March 2020, May 2020, August 2020, August 2021) to all practicing ophthalmologists in PR (N ≈ 200), capturing data on closures, patient volume, personal protective equipment (PPE) access, telemedicine use, and financial relief. **Results**: Survey responses ranged from 41% (*n* = 81) to 56% (*n* = 111). By March 2020, 22% (24/111) of respondents closed their offices. By May 2020, 20% (19/93) of respondents maintained a closed office, while 89% (64/72) of open offices reported seeing less than 25% of their usual patient volume. Access to PPE was a challenge, with 59% (65/111) reporting difficulty obtaining N95 masks in March 2020. Telemedicine usage increased initially, peaking in May 2020 and declining in July 2020. By August 2021, all respondents were fully vaccinated and most practices returned to pre-pandemic levels. Overall, 86% (70/81) of respondents found the surveys to be useful for navigating practice changes during the pandemic. **Conclusions**: PR ophthalmologists showed adaptability during the COVID-19 pandemic to maintain care given limited resources. Guidelines from professional organizations and real time surveys play an important role in future crisis preparedness.

## 1. Introduction

Healthcare providers faced the complex challenge of ensuring patient care and upholding public health protocols in the midst of uncertainty during the COVID-19 pandemic. The ophthalmology field particularly had major disadvantages. Hospitals and critical care units received priority in personal protective equipment (PPE), leaving ophthalmology practices to ration supplies and delay nonurgent care [1,2,3]. With its unique socio-economic, geographic, and political environment, Puerto Rico (PR) was exposed to a distinct set of vulnerabilities. Its status as a United States (U.S.) territory, combined with high rates of poverty, relative geographic isolation, and a limited supply of both medical resources and personnel, complicated efforts to develop timely and effective emergency responses. This included delays in the acquisition of necessary PPE as compared to states in the contiguous U.S. [4]. Further, the COVID-19 infection rate in certain rural areas of PR were as high as those found in urban areas [5], countering general trends that urban areas had higher case rates [6]. However, PR has demonstrated resilience in the face of previous public health crises, most notably during the aftermath of Hurricane Maria in 2017 and a series of earthquakes in 2020 [7].

Rapid changes in laws and regulations in PR between March 2020 and August 2021 further shaped the structural conditions surrounding the pandemic. On 15 March 2020, PR declared a state of emergency in view of the rapid rise in cases of COVID-19 [8], which caused an unprecedented disruption of health care services [5]. Further, the Centers for Medicare and Medicaid Services and the American Academy of Ophthalmology (AAO) issued national guidance on the delay of elective surgery to conserve health resources and to reduce the transmission probability of COVID-19 [9,10]. Policy and guideline changes compelled ophthalmologic practices across the island to redefine standards of care, which continued to evolve through different phases of the pandemic. Updated standards considered the need to provide essential ophthalmic care while minimizing the risk of transmission of the virus [1].

By April 2020, multiple COVID-19 financial relief initiatives became available to assist practices in the U.S. with maintaining financial viability and supporting their staff [11,12]. The Paycheck Protection Program provided forgivable loans for payroll expenses. The Economic Injury Disaster Loan offered low-interest loans to compensate for economic losses. Lastly, the Health and Human Services Automatic Provider Relief Funds helped health care providers offset lost revenue and additional expenses [11]. By July 2020, loosening of restrictive measures allowed ophthalmologists to reopen clinics and resume elective surgeries [13].

The impetus for the surveys used in this current study arose from unclear guidelines by the local government, health departments, and medical societies at the onset of the COVID-19 pandemic. Large national medical professional organizations, such as the AAO [14] and the American Society of Retina Surgeons (ASRS) [15], rapidly launched surveys to assess changing practice patterns in response to the pandemic and guide their communities. The senior author, who is a practicing ophthalmologist in PR, identified a gap in localized, context-specific information necessary to guide clinical practice decisions. Thus, a series of surveys were designed to gather information at several critical junctures of the pandemic to help tailor protocols and encourage communication, collegiality, and community among ophthalmologists in PR. Rapid dissemination of survey results to the respondents intended to empower practitioners to make timely, knowledgeable choices. While the study surveys were not designed with formal research methodology in mind, they examine how ophthalmologists in PR adapted their practice patterns in response to the state of emergency, delays in elective surgeries, relief measures, and the introduction of COVID-19 vaccines. The authors also intended to assess the utility of surveys as a form of communication during the pandemic to aid the professional community adapt to the evolving and ambiguous practice guidelines. Identifying successful strategies, challenges, and opportunities for improvement may help expand the knowledge base and inform future preparedness efforts with the goal of ultimately enhancing the quality and accessibility of ophthalmic care in PR. Furthermore, identifying the role of survey tools with rapid feedback in crisis situations could aid medical communities in PR and elsewhere.

## 2. Materials and Methods

**Figure 1:**
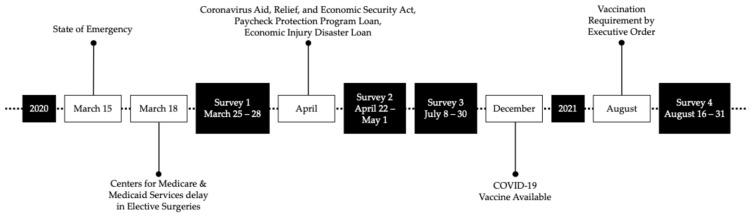
Survey distribution timing in correlation with critical points during the COVID-19 pandemic.

This study employed a series of cross-sectional surveys across the island to investigate the adjustments made by ophthalmologists in PR in response to the COVID-19 pandemic. Anonymous surveys were disseminated using SurveyMonkey at four distinct intervals: Survey 1 (25–28 March 2020), Survey 2 (22 April–1 May 2020), Survey 3 (8–30 July 2020), and Survey 4 (16–31 August 2021). Each survey was strategically timed to coincide with significant milestones during the COVID-19 pandemic, capturing crucial stages and developments in the evolving crisis (Figure 1). Survey 1 focused on early-stage responses such as reducing operational capacity, purchasing PPE, and adopting telemedicine. Survey 2 expanded on Survey 1 questions to encompass questions about financial relief and COVID-19 screening procedures. Survey 3 further explored efforts to restore pre-COVID operations while continuing to adapt to ongoing COVID-related challenges. Lastly, Survey 4 was created in response to a new local PR executive orders requiring vaccination to enter private establishments [16,17], resulting in ambiguity for medical offices. Thus, the survey examined protocols around patient entry, screening, and managing positive cases, and usefulness of the surveys. All surveys consisted of both closed-ended and open-ended questions, including questions about demographics (age group, years of practice) and practice information (geographic health district, subspecialty, practice size). The questions were designed by three authors (LA, JC, HJ) based on their personal experiences, concerns discussed among ophthalmologists in PR in various chats, and concurrent surveys by the AAO and the ASRS [13,15].

All ophthalmologists practicing in PR were invited to participate (N ≈ 200) based on the National Provider Identifier Database [18] and information from the Sociedad Puertorriqueña de Oftalmología through personal communication. Convenient samples of ophthalmologists were recruited at each survey period via outreach through multiple channels, including the Sociedad Puertorriqueña de Oftalmología email listserv and various ophthalmology WhatsApp chats (e.g., Eye Care Puerto Rico and Women in Ophthalmology), an instant message application used frequently among ophthalmologists in PR. A public link to the study survey was provided in email and text message exchanges. Ophthalmologists who were actively practicing in PR and were not in residency or fellowship training were included in the sample.

Descriptive analyses were conducted to describe characteristics of the sample and practice patterns at each survey period. Missing values were retained in the dataset. All analyses were conducted using STATA/BE Version 18.

The study was approved by Sterling (Atlanta, GA, USA) Institutional Review Board (blinded for review purposes). The researchers adhered to the tenets of the Declaration of Helsinki.

## 3. Results

### 3.1. Sample Characteristics

Survey response rates ranged from 41% to 56% of the estimated 200 ophthalmologists as reported by National Provider Identifier Database [18]. Across all surveys, most participants were 35–64 years old, were comprehensive ophthalmologists, located in the metropolitan area, and were solo practitioners (Table 1). In the last survey, 86% (70/81) of participants found the surveys to be useful.

### 3.2. Adaptations in Ophthalmology Clinical Practices

Clinic closures and patient volume varied during the COVID-19 pandemic (Table 2). By March 2020, 22% (24/111) of participants reported that their offices were closed after Governor Wanda Vázquez Garced declared an executive ordinance on 15 March. Of the remaining 77% (86/111) of participants whose offices were open, 64% (55/86) had 25% or less of the usual patient volume, 3% (3/86) had 50% of the usual patient volume, and 33% (28/86) had missing patient volumes. By May 2020, 20% (19/93) of participants reported that their offices were closed. Of the 77% (72/93) which were open, 89% (64/72) were seeing 25% or less of their usual patient volume. Further, 88% (63/72) only saw emergency and urgent visits, while 13% (9/72) were open for all patient care. By July 2020, 100% (82/82) of participants had their offices open. Many participants reported having 75% of their usual patient volume by July 2020 [61% (50/82)] and August 2021 [48% (39/81)]. Specific to surgical operations, most participants were not operating in March 2020 [81% (90/111)]. Among those who were operating at that time, as high as 95% (20/21) of ophthalmologists were available for only emergency cases. During this time period, 71% (75/105) of physicians reported that operating rooms were available for only emergency cases while 6% (6/105) reported they were available for all cases. By May 2020, only 33% (25/75) of self-reported ophthalmic surgeons were not operating and 100% (50/50) of those available to operate were operating only on emergency cases. The availability of operating rooms was reduced to 69% (55/80) for emergency cases and 3% (2/80) for all cases. A small percent of self-reported ophthalmic surgeons continued to not operate by July 2020 [10% (7/71)], but inclusion of elective cases increased at that time [77% (55/71)] with a small percentage operating only on emergency cases [13% (9/71)].

When asked why their practice was closed or only open for emergency and urgent cases in May 2020, many respondents reported following the guidelines of medical organizations [72% (59/82)], such as the AAO, Sociedad Puertoriqueno de Oftalmologia and Colegio de Médicos-Cirujanos de PR, and having concerns of not wanting to put themselves, their staff and patients at risk of infection from COVID-19 [68% (56/82)]. For those who chose to open or reopen their practice at that time, 83% (60/72) felt a moral obligation to care for their patients and community, 21% (15/72) felt a financial obligation, and 11% (8/72) felt that they were at minimal risk for COVID-19.

The use of telemedicine fluctuated between March and July 2020. About 19% (21/111) of participants utilized telemedicine by March, increasing to 38% (35/93) by May and to 22% (18/82) in July 2020. The number of participants who wanted to use telemedicine but did not know how was reported at 16% (18/111) by March, 10% (9/93) by May and none by July 2020.

### 3.3. PPE Usage

**Figure 2:**
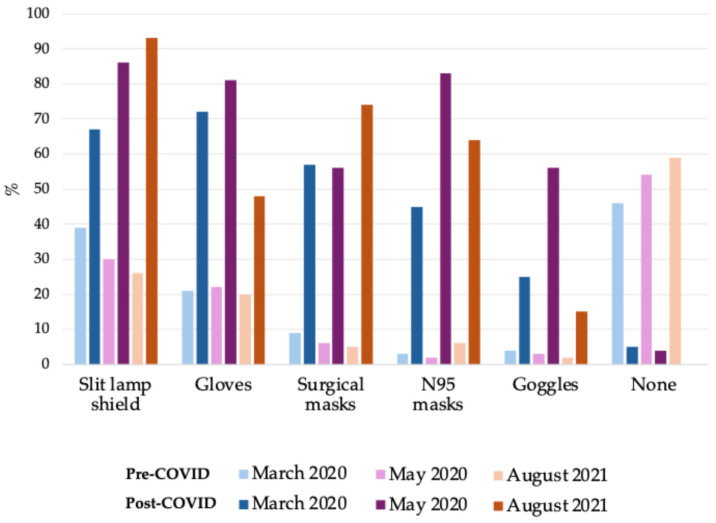
Office use of personal protective equipment before and during the COVID-19 pandemic asked at critical time points: March 2020 (*n* = 111), May 2020 (*n* = 93), and August 2021 (*n* = 81); not available in Survey 3 (July 2020). The questions allowed for multiple selections.

Figure 2 shows the use of PPE before and during the pandemic. By March and May 2020, few participants reported having used gloves before the pandemic (21–22%), while a majority reported using gloves during the pandemic (72–81%). By August 2021, use of gloves reduced to 48% (39/81) of participants. Based on responses from three surveys (March 2020, May 2020, August 2021), the use of surgical and N95 masks was 5–9% and 2–6%, respectively, pre-pandemic and rose steadily during the pandemic. By March 2020, use of surgical and N95 masks increased to 57% and 45%, respectively, then 56% and 83% by May 2020, to 74% and 64% by August 2021. Of note, is the sharp rise in slit lamp shields from 26–39% pre-pandemic to 67–93% during the pandemic. By March 2020, when asked about access to purchasing PPE, ophthalmologists reported wanting but not being able to obtain N95 masks [59% (65/111)], disinfectants [28% (31/111)], surgical masks [18% (20/111)] and gloves [14% (16/111)]. In the same time period, 38% (42/111) of ophthalmologists who had ordered N95 masks since 16 March 2020 reported paying $4 or more for N95 masks, and a higher proportion was observed by May 2020 [55% (33/60)].

### 3.4. Federal Financial Assistance

Survey respondents reported on utilization of federal assistance programs in May and July 2020 (Table 3). By May 2020, 18% (17/93) of participants were approved for the Paycheck Protection Program Loan, while 57% (53/93) were still applying for it. By July 2020, 79% (65/82) of participants had received the Paycheck Protection Program Loan. The Economic Injury Disaster Loan was less utilized in May 2020 and July 2020, with 22–26% of participants not knowing about it and 38–40% of participants not being interested in it. Many participants received the U.S. Health and Human Services Stimulus Grant by May 2020 [71% (66/93)] and even more so by July 2020 [78% (64/82)].

### 3.5. COVID-19 Vaccination, Screening, and Control

Within a few weeks after the Governor’s executive orders in August 2021 requiring proof of vaccination to enter private establishments [16,17], 51% (41/81) of respondents required patients to show proof of vaccination or a negative COVID-19 test to enter their office, 28% (23/81) reported planning on instituting this policy in the near future and 21% (17/81) had no intention of instituting this policy. By August 2021, 100% (81/81) of participants reported that they were fully vaccinated and 93% (75/81) had not tested positive for COVID-19. In the same time period, a majority of participants reported that all their clinical staff were vaccinated against COVID-19 [80% (65/81)]. Among the 91% (74/81) of ophthalmologists who always or sometimes asked their patients or parents of patients under the age 12 for vaccination status, 46% (34/74) had more than 90% of their patient volume vaccinated, while 47% (35/74) had 50–90% of their patient volume vaccinated. If a staff member tested positive for COVID-19, 49% (40/81) of respondents required quarantine only among infected and exposed staff and 32% (26/81) closed down their office until all other staff members tested negative. For surgical patients, 41% (33/81) of respondents required proof of vaccination or a negative polymerase chain reaction COVID-19 test prior to surgery, while 21% (17/81) required proof of a negative polymerase chain reaction COVID-19 test regardless of vaccination status.

## 4. Discussion

The current study distributed surveys among ophthalmologists in PR during the COVID-19 pandemic, which yielded high response rates ranging from 41% to 56%. Respondents reported that professional organizations, such as the AAO, Sociedad Puertorriqueña de Oftalmología, and Colegio de Médicos-Cirujanos de PR, played an essential role in guiding practice patterns during the pandemic. Rapid responses to the pandemic by PR ophthalmologists, including closing their offices, underscored their commitment to public health and patient care. The surveys highlighted the challenges and cost of acquiring PPE, specifically N95 masks. Additionally, telemedicine usage increased temporarily but declined as clinics reopened. There was widespread adoption of federal economic relief aimed to help sustain ophthalmology practices during the pandemic. About 86% of respondents in the final survey reported that the surveys were helpful, demonstrating the utility of rapid analysis of survey data in effectively facilitating knowledge-sharing within communities of physicians. Simple surveys such as those used in this study serve as a promising tool in fostering a sense of camaraderie during times of uncertainty and guiding future public health planning.

### 4.1. Demographic Context and Its Implications

The present study demonstrated higher participation (41–56%) compared to similar surveys conducted by the ASRS and AAO, which reported a 20–39% [15] and 7–16% response rate, respectively [13,14,19]. Survey response rates have previously been observed to be high when affiliated with a professional organization or among physicians with personal ties [20]. Strong engagement in our study may have reflected a heightened interest in sharing experiences, willingness to provide insights of their local community, and the close-knit ophthalmology community of PR. Further, continued participation in each successive survey may be credited to the prompt availability of findings. The 48–59% of solo practitioners in the study sample, a proportion notably higher than the estimated 26% of ophthalmologists in solo practice nationally [21], may have particularly benefitted from the surveys. Solo practitioners were previously identified to have faced unique challenges during the pandemic, including limited resources, financial pressures, and the need to implement telemedicine without the support of a group practice [22,23]. Our success with surveys demonstrates its effectiveness as a feasible, low-cost method to facilitate real-time communication within the ophthalmology community, potentially fostering a sense of shared experience and support, reducing isolation and encouraging collective problem-solving [24]. Other medical specialties may benefit from leveraging surveys as part of their emergency response strategies.

The demographics of respondents revealed a trend towards older practitioners, with 20–28% aged 55–64 and 16–24% over 65 years old. This trend is consistent with broader data from the U.S. Department of Health and Human Services, which showed that a significant portion of the ophthalmology workforce in the U.S. was over the age of 55 [25]. This skew is crucial for interpreting the survey results, as older ophthalmologists, with their extensive experience and potential health concerns, might have been more cautious in their approach to pandemic-related practice changes. A cross-sectional study conducted among family physicians in Ontario, Canada found that many older physicians considered early retirement during the pandemic due to the increased risks associated with age and the stress of adapting to new safety protocols [26].

### 4.2. Guidance and Response Time

Study findings showed rapid responses by the ophthalmology community in PR after the Governor’s executive stay-at-home orders on 15 March 2020. About 22% (24/111) of respondents closed their practices by March 2020. Further, 71% (79/111) reported their office closed or open and seeing less than 25% volume vs. 95% reported in April by the AAO [19]. The modest disparity in patient volume rates may be due to the large number of missing responses in our survey among ophthalmologists reporting their patient volume at an open office [33%, 28/86]. Low volumes in the early phases of the pandemic were also reported by the Pan-American Association of Ophthalmology where 67% of the participating countries had more than 80% reduction in patient volume [27]. A report by the Commonwealth Fund found that ophthalmology outpatient practices witnessed the highest decline of outpatient visits (greater than 70%) than any other medical specialty by April 2020 [28]. Meanwhile, the ASRS reported only 40% [15] of respondents were closed or seeing less than 25% of volume in March, which could reflect the more urgent nature of retinal disease often requiring monthly intravitreal injections to prevent blindness. By May, the AAO and ASRS reported a reduction in respondents reporting less than 25% volume to 40% and 10%, respectively, versus 89% in this study. The difference could be due to the strict shelter-in-place measures in PR that persisted in May [29,30].

Findings related to the re-establishment of clinical capacity and adherence to new public health measures during the later phases of the pandemic were consistent with broader national trends [14,15]. By July 2020, 61% (50/82) of respondents in our sample increased their clinic’s patient volume to at least 75% of usual volume and most extended care to all patient types. Further, all respondents reported that their clinics had resumed operations by August 2021, of which 85% (69/81) reached 75–100% of their pre-pandemic patient volumes.

Ethical considerations played a vital role in the decision to reduce clinical capacity among PR ophthalmologists. Many respondents in our study adjusted their services based on ethical or personal decisions to avoid exposing themselves or others to the risk of COVID-19 infection. On the other hand, the majority of those who continued to provide some level of patient care felt a moral obligation to their patients and the community at large. Similar ethical dilemmas have been widely reported by other healthcare providers in the U.S. and other countries [31]. Healthcare systems and professional societies should consider integrating mental health support, public health ethic training, and peer discussion in crisis response plans to minimize possible psychological strain. The AAO, Sociedad Puertorriqueña de Oftalmología, and Colegio de Médicos-Cirujanos de PR may be well-positioned to adopt these strategies given their influence in guiding practice patterns among ophthalmologists in our study.

By August 2021, 100% (81/81) of respondents were fully vaccinated, closely paralleling the estimated 96% vaccination rate among members of the AAO [32] during this time. Additionally, 80% (65/81) of the respondents’ clinical staff were fully vaccinated against COVID-19, comparable to the 78% of U.S. ophthalmology staff reported to have received their full vaccination series [32].

### 4.3. Personal Protective Equipment Utilization and Challenges

Our findings support global trends in PPE usage among practicing ophthalmologists during the COVID-19 pandemic. In the current study, the use of surgical and N95 masks was minimal prior to the pandemic and increased up to 74% and 83% during the pandemic, respectively. Two studies conducted globally found high proportions of ophthalmologists (92–97%) who used face masks as a preventative measure against COVID-19 [33,34]. These responses reflect multiple masking recommendations by the AAO [35] and other researchers [36,37] as ophthalmologists were especially vulnerable to COVID-19 transmission given the proximity to patients’ faces during the ophthalmic exam and the potential viral transmission via tears and the conjunctiva [38,39].

Access to PPE, was found to be a significant challenge in our study. Many ophthalmologists who participated in our March 2020 survey reported difficulties in obtaining N95 masks, surgical masks, gloves, and disinfectants. Similar findings were also described by the ASRS where half of U.S. respondents reported difficulty accessing N95 or surgical masks in March 2020 [15]. Participants in this study noted the high costs of N95 masks, with some paying at least $4 per mask. The reported costs were comparable to the jump in prices for N95 respirators–from $0.98 to $7.4–revealed by a hospital in Illinois [40]. Improving PPE supply chains and implementing safeguards against price gouging should be considered in preparing for future public health crises, especially in isolated locations such as PR.

### 4.4. Telemedicine

The COVID-19 pandemic necessitated a swift pivot to telemedicine across many medical specialties, including ophthalmology, to maintain patient care while adhering to social distancing guidelines. Approximately 38% (35/93) of ophthalmologists in our study utilized telemedicine by May 2020–a prompt response, though below the 54% reported by the AAO [13]. By July 2020, the use of telemedicine declined among our participants, coinciding with the reopening of clinics around this time and supporting similar findings of the AAO [13] and University of California San Francisco [41]. While telemedicine was a viable interim solution, it did not secure a lasting place in the practice of ophthalmology in PR. The unique requirements of ophthalmic care, such as the need for equipment for a physical examination (e.g., slit lamp and ophthalmoscope) and specialized in-person diagnostic tests (e.g., visual field testing and optical coherence tomography), present significant challenges for remote care. Reimbursement challenges for telemedicine were also suggested as a barrier for long term use [42]. Notably, the increased use of telemedicine by ophthalmologists during the pandemic helped increase their confidence for future use [43]. As home-based ophthalmic diagnostic imaging technology improves, telemedicine in ophthalmology may become more widely adopted in the future [44].

### 4.5. Financial Assistance

Ophthalmologists, like many healthcare professionals, faced unprecedented operational and financial challenges during the COVID-19 pandemic [19,45]. Federal assistance programs, including the Economic Injury Disaster Loan, Paycheck Protection Program Loan, and Health and Human Services grants, played pivotal roles in supporting ophthalmology practices during this tumultuous period [19,46].

About 71–78% of respondents reported receiving a Health and Human Services Stimulus Grant by May and July 2020, which provided automatic deposits of funds without the need of an application process. This experience is consistent with the broader U.S. context, where the Health and Human Services distributed billions of dollars in Provider Relief Fund payments to healthcare providers nationwide, helping practices maintain operations and support staff during the peak of the pandemic [46]. The Paycheck Protection Program Loan, a fully reimbursable loan designed to help businesses keep their workforce employed during the pandemic, may have served as a lifeline for ophthalmology practices against the downturn in patient volume and revenue. Its broad uptake by July 2020 was demonstrated in both our study (79%) and the AAO’s (88%) [14]. The Economic Injury Disaster Loan program offered low-interest loans and was not commonly utilized by ophthalmologists in this study (19–23%). This might be attributed to the need to repay the Economic Injury Disaster Loan as compared to the potentially forgivable Paycheck Protection Program Loans. Future financial assistance policies could prioritize forgivable loans aimed at retaining employees over traditional loans.

### 4.6. Limitations

Several factors restrict the scope of this current research. Firstly, data was collected via self-administered online surveys. Self-reports are commonly known to be susceptible to recall and social desirability biases. Although anonymity was championed throughout data collection, sensitive questions about vaccination status, ever testing positive for COVID-19, and personal reasons for temporarily reducing operational capacity may have influenced participant responses. Secondly, the surveys used in this analysis were adapted to contexts of the time period during which they were disseminated. Thus, several questions appeared exclusively in certain surveys, limiting the ability to conduct comparisons across time points. Lastly, the cross-sectional design provides only snapshots of critical time points during the pandemic. Future studies may wish to conduct in-depth interviews with ophthalmologists in PR to capture firsthand accounts of how decisions were made under uncertainty and evolving conditions. These granular insights could help reveal constraints that were overlooked in the study surveys.

### 4.7. Lessons Learned and Future Directions

The COVID-19 pandemic resulted in barriers and delays to ophthalmic care resulting in vision loss [15,47]. Ophthalmic practices attempted to rapidly adapt to changing guidelines to contain the pandemic while providing ophthalmic care. The pandemic acted as a catalyst for experimenting with changes in healthcare delivery while maintaining safe practices. Although some of these changes, such as telemedicine, were not sustained [48], they served to equip physicians to reimplement them if needed in the future. Our series of surveys conducted among ophthalmologists in PR captured the evolution of practice patterns at various time points, the difficulty in obtaining and affording PPE, the fleeting role of telemedicine adoption, participation in loan programs, and the importance of guidance from professional organizations. Furthermore, it documented the utility of surveys as a tool for data collection and information sharing among healthcare professionals in this population. Such surveys can facilitate peer-to-peer learning, modify standards of care that can adapt to evolving challenges, and foster community to aid the medical community navigate future crises.

## Figures and Tables

**Table 1:** Sample characteristics.

	Survey							
	1 March 2020		2 May 2020		3 July 2020		4 August 2021	
	*(n* = 111)		(*n* = 93)		(*n* = 82)		(*n* = 81)	
	No	%	No	%	No	%	No	%
Age Group								
25–34	4	4	6	6	0	0	5	6
35–44	29	26	20	22	18	22	17	21
45–54	31	28	26	28	24	29	21	26
55–64	28	25	19	20	21	26	23	28
65+	18	16	22	24	19	23	14	17
Subspecialty ^a^								
Comprehensive	67	60	59	63	46	56	56	69
Medical and Surgical Retina	20	18	16	17	17	21	18	22
Glaucoma	15	14	14	15	11	13	10	12
Cornea	12	11	15	16	17	21	7	9
Medical Retina	7	6	*	*	*	*	5	6
Oculoplastics	5	5	5	5	6	7	5	6
Pediatric or Strabismus	*	*	5	5	*	*	*	*
Neuro-ophthalmology	*	*	*	*	*	*	*	*
Uveitis	*	*	*	*	*	*	*	*
Geographic health district ^a^								
Metropolitan	50	45	46	49	41	50	40	49
Mayagüez	14	13	9	10	11	13	6	7
Arecibo	13	12	13	14	16	20	11	14
Bayamón	12	11	12	13	13	16	13	16
Caguas	10	9	14	15	7	9	9	11
Ponce	8	7	8	9	7	9	7	9
Fajardo	*	*	6	6	*	*	*	*
Practice type ^b^								
Solo Practitioner	66	59	55	59	47	57	39	48
Small Group (<5)	27	24	25	27	18	22	28	35
Large Group (≥5)	18	16	13	14	11	13	13	16
University Setting	17	15	12	13	*	*	7	9
Veterans Hospital	5	5	5	5	5	6	*	*
Fondo	*	*	*	*	*	*	–	–

* Data suppressed for cell counts < 5 to protect confidentiality. – Was not asked in the survey. ^a^ Select all that apply. ^b^ Select all that apply except in Survey 3.

**Table 2:** Ophthalmology offices after the executive ordinance on 15 March 2020.

	Survey					
	1 March 2020		2 May 2020		3 July 2020	
	*(n* = 111)		(*n* = 93)		(*n* = 82)	
	No	%	No	%	No	%
Office hours						
Open	86	77	72	77	82	100
Emergency only	–	–	63	88	4	5
All visits	–	–	9	13	78	95
Closed	24	22	19	20	0	0
Missing	1	<1	2	2	0	0
Patient volume if open						
25% or less of usual	55	64	64	89	5	6
50% of usual	3	3	5	7	23	28
75% of usual	0	0	0	0	50	61
100% of usual	0	0	0	0	3	4
Missing	28	33	3	4	1	1

– Was not asked in the survey.

**Table 3:** Utilization of federal assistance programs.

	Survey			
	2 May 2020		3 July 2020	
	(*n* = 93)		(*n* = 82)	
	No	%	No	%
Applied for a Paycheck Protection Program Loan				
Applying	53	57	2	2
Approved	17	18	65	79
Ineligible because of other reasons	10	11	7	9
Heard of loan, would like more information	5	5	1	1
Ineligible because office is closed	3	3	0	0
Do not know about the loan	1	1	2	2
Applied for Economic Injury Disaster Loan				
Not interested	35	38	33	40
Do not know about the loan	24	26	18	22
Applied/applying	18	19	19	23
Heard of loan, would like more information	9	10	3	4
Ineligible	5	5	7	9
Received Health and Human Services Stimulus Grant				
Received	66	71	64	78
Not received	17	18	9	11
Do not know if I, my group, or my employer received	8	9	6	7
Do not know about the grant	2	2	2	2

## Data Availability

The data presented in this study are available on request from the corresponding author due to privacy reasons.

## Abbreviations

The following abbreviations are used in this manuscript:

PR	Puerto Rico
U.S.	United States
